# Supplementary material for: Resolving the full spectrum of human genome variation using Linked-Reads
Source: Genome Res. 2019 Apr;29(4):635–45. doi: 10.1101/gr.234443.118 (PMC6442396; doi:10.1101/gr.234443.118)
Supplement: Supplemental Material [file supp_gr.234443.118_Supplemental_longranger-2.2.zip › longranger-2.2.tar.bz2!/lr2.2-public/lib/stan/cmdstan/stan/doc/api/html/logistic_8hpp_source.html]

Stan: src/stan/prob/distributions/univariate/continuous/logistic.hpp Source File


|  |  |
| --- | --- |
|  | Stan  2.5.0  probability, sampling & optimization |


- Main Page
- Related Pages
- Namespaces
- Classes
- Files

- File List
- File Members

All Classes Namespaces Files Functions Variables Typedefs Enumerations Enumerator Friends Macros Pages

- src
- stan
- prob
- distributions
- univariate
- continuous

logistic.hpp

Go to the documentation of this file.

1 #ifndef STAN\_\_PROB\_\_DISTRIBUTIONS\_\_UNIVARIATE\_\_CONTINUOUS\_\_LOGISTIC\_HPP

2 #define STAN\_\_PROB\_\_DISTRIBUTIONS\_\_UNIVARIATE\_\_CONTINUOUS\_\_LOGISTIC\_HPP

3

4 #include <boost/random/exponential\_distribution.hpp>

5 #include <boost/random/variate\_generator.hpp>

6

7 #include <stan/agrad/partials\_vari.hpp>

8 #include <stan/math/error\_handling/check\_not\_nan.hpp>

9 #include <stan/math/error\_handling/check\_positive\_finite.hpp>

10 #include <stan/math/error\_handling/check\_finite.hpp>

11 #include <stan/math/error\_handling/check\_consistent\_sizes.hpp>

12 #include <stan/math/constants.hpp>

13 #include <stan/math/functions/value\_of.hpp>

14 #include <stan/math/functions/log1p.hpp>

15 #include <stan/meta/traits.hpp>

16 #include <stan/prob/constants.hpp>

17 #include <stan/prob/traits.hpp>

18

19 namespace stan {

20  namespace prob {

21

22  // Logistic(y|mu,sigma) [sigma > 0]

23  // FIXME: document

24  template <bool propto,

25  typename T\_y, typename T\_loc, typename T\_scale>

26  typename return\_type<T\_y,T\_loc,T\_scale>::type

27  logistic\_log(const T\_y& y, const T\_loc& mu, const T\_scale& sigma) {

28  static const char\* function = "stan::prob::logistic\_log(%1%)";

29

30  using stan::math::check\_positive\_finite;

31  using stan::math::check\_finite;

32  using stan::math::check\_consistent\_sizes;

33  using stan::math::value\_of;

34  using stan::prob::include\_summand;

35

36  // check if any vectors are zero length

37  if (!(stan::length(y)

38  && stan::length(mu)

39  && stan::length(sigma)))

40  return 0.0;

41

42

43  // set up return value accumulator

44  double logp(0.0);

45

46  // validate args (here done over var, which should be OK)

47  check\_finite(function, y, "Random variable", &logp);

48  check\_finite(function, mu, "Location parameter", &logp);

49  check\_positive\_finite(function, sigma, "Scale parameter", &logp);

50  check\_consistent\_sizes(function,

51  y,mu,sigma,

52  "Random variable","Location parameter",

53  "Scale parameter",

54  &logp);

55

56  // check if no variables are involved and prop-to

57  if (!include\_summand<propto,T\_y,T\_loc,T\_scale>::value)

58  return 0.0;

59

60

61  // set up template expressions wrapping scalars into vector views

62  agrad::OperandsAndPartials<T\_y, T\_loc, T\_scale>

63  operands\_and\_partials(y, mu, sigma);

64

65  VectorView<const T\_y> y\_vec(y);

66  VectorView<const T\_loc> mu\_vec(mu);

67  VectorView<const T\_scale> sigma\_vec(sigma);

68  size\_t N = max\_size(y, mu, sigma);

69

70  DoubleVectorView<true,is\_vector<T\_scale>::value>

71  inv\_sigma(length(sigma));

72  DoubleVectorView<include\_summand<propto,T\_scale>::value,

73  is\_vector<T\_scale>::value> log\_sigma(length(sigma));

74  for (size\_t i = 0; i < length(sigma); i++) {

75  inv\_sigma[i] = 1.0 / value\_of(sigma\_vec[i]);

76  if (include\_summand<propto,T\_scale>::value)

77  log\_sigma[i] = log(value\_of(sigma\_vec[i]));

78  }

79

80  DoubleVectorView<!is\_constant\_struct<T\_loc>::value,

81  is\_vector<T\_loc>::value || is\_vector<T\_scale>::value>

82  exp\_mu\_div\_sigma(max\_size(mu,sigma));

83  DoubleVectorView<!is\_constant\_struct<T\_loc>::value,

84  is\_vector<T\_y>::value || is\_vector<T\_scale>::value>

85  exp\_y\_div\_sigma(max\_size(y,sigma));

86  if (!is\_constant\_struct<T\_loc>::value) {

87  for (size\_t n = 0; n < max\_size(mu,sigma); n++)

88  exp\_mu\_div\_sigma[n] = exp(value\_of(mu\_vec[n])

89  / value\_of(sigma\_vec[n]));

90  for (size\_t n = 0; n < max\_size(y,sigma); n++)

91  exp\_y\_div\_sigma[n] = exp(value\_of(y\_vec[n])

92  / value\_of(sigma\_vec[n]));

93  }

94

95  using stan::math::log1p;

96  for (size\_t n = 0; n < N; n++) {

97  const double y\_dbl = value\_of(y\_vec[n]);

98  const double mu\_dbl = value\_of(mu\_vec[n]);

99

100  const double y\_minus\_mu = y\_dbl - mu\_dbl;

101  const double y\_minus\_mu\_div\_sigma = y\_minus\_mu \* inv\_sigma[n];

102  double exp\_m\_y\_minus\_mu\_div\_sigma(0);

103  if (include\_summand<propto,T\_y,T\_loc,T\_scale>::value)

104  exp\_m\_y\_minus\_mu\_div\_sigma = exp(-y\_minus\_mu\_div\_sigma);

105  double inv\_1p\_exp\_y\_minus\_mu\_div\_sigma(0);

106  if (!is\_constant\_struct<T\_y>::value

107  || !is\_constant\_struct<T\_scale>::value)

108  inv\_1p\_exp\_y\_minus\_mu\_div\_sigma = 1 / (1 + exp(y\_minus\_mu\_div\_sigma));

109

110  if (include\_summand<propto,T\_y,T\_loc,T\_scale>::value)

111  logp -= y\_minus\_mu\_div\_sigma;

112  if (include\_summand<propto,T\_scale>::value)

113  logp -= log\_sigma[n];

114  if (include\_summand<propto,T\_y,T\_loc,T\_scale>::value)

115  logp -= 2.0 \* log1p(exp\_m\_y\_minus\_mu\_div\_sigma);

116

117  if (!is\_constant\_struct<T\_y>::value)

118  operands\_and\_partials.d\_x1[n]

119  += (2 \* inv\_1p\_exp\_y\_minus\_mu\_div\_sigma - 1) \* inv\_sigma[n];

120  if (!is\_constant\_struct<T\_loc>::value)

121  operands\_and\_partials.d\_x2[n] +=

122  (1 - 2 \* exp\_mu\_div\_sigma[n] / (exp\_mu\_div\_sigma[n]

123  + exp\_y\_div\_sigma[n]))

124  \* inv\_sigma[n];

125  if (!is\_constant\_struct<T\_scale>::value)

126  operands\_and\_partials.d\_x3[n] +=

127  ((1 - 2 \* inv\_1p\_exp\_y\_minus\_mu\_div\_sigma)

128  \*y\_minus\_mu\*inv\_sigma[n] - 1) \* inv\_sigma[n];

129  }

130  return operands\_and\_partials.to\_var(logp);

131  }

132

133  template <typename T\_y, typename T\_loc, typename T\_scale>

134  inline

135  typename return\_type<T\_y,T\_loc,T\_scale>::type

136  logistic\_log(const T\_y& y, const T\_loc& mu, const T\_scale& sigma) {

137  return logistic\_log<false>(y,mu,sigma);

138  }

139

140  // Logistic(y|mu,sigma) [sigma > 0]

141  template <typename T\_y, typename T\_loc, typename T\_scale>

142  typename return\_type<T\_y, T\_loc, T\_scale>::type

143  logistic\_cdf(const T\_y& y, const T\_loc& mu, const T\_scale& sigma) {

144

145  // Size checks

146  if ( !( stan::length(y) && stan::length(mu)

147  && stan::length(sigma) ) )

148  return 1.0;

149

150  // Error checks

151  static const char\* function = "stan::prob::logistic\_cdf(%1%)";

152

153  using stan::math::check\_not\_nan;

154  using stan::math::check\_positive\_finite;

155  using stan::math::check\_finite;

156  using stan::math::check\_consistent\_sizes;

157  using stan::math::value\_of;

158  using boost::math::tools::promote\_args;

159

160  double P(1.0);

161

162  check\_not\_nan(function, y, "Random variable", &P);

163  check\_finite(function, mu, "Location parameter", &P);

164  check\_positive\_finite(function, sigma, "Scale parameter", &P);

165  check\_consistent\_sizes(function, y, mu, sigma,

166  "Random variable", "Location parameter",

167  "Scale parameter", &P);

168

169  // Wrap arguments in vectors

170  VectorView<const T\_y> y\_vec(y);

171  VectorView<const T\_loc> mu\_vec(mu);

172  VectorView<const T\_scale> sigma\_vec(sigma);

173  size\_t N = max\_size(y, mu, sigma);

174

175  agrad::OperandsAndPartials<T\_y, T\_loc, T\_scale>

176  operands\_and\_partials(y, mu, sigma);

177

178  // Explicit return for extreme values

179  // The gradients are technically ill-defined, but treated as zero

180

181  for (size\_t i = 0; i < stan::length(y); i++) {

182  if (value\_of(y\_vec[i]) == -std::numeric\_limits<double>::infinity())

183  return operands\_and\_partials.to\_var(0.0);

184  }

185

186  // Compute vectorized CDF and its gradients

187  for (size\_t n = 0; n < N; n++) {

188

189  // Explicit results for extreme values

190  // The gradients are technically ill-defined, but treated as zero

191  if (value\_of(y\_vec[n]) == std::numeric\_limits<double>::infinity()) {

192  continue;

193  }

194

195  // Pull out values

196  const double y\_dbl = value\_of(y\_vec[n]);

197  const double mu\_dbl = value\_of(mu\_vec[n]);

198  const double sigma\_dbl = value\_of(sigma\_vec[n]);

199  const double sigma\_inv\_vec = 1.0 / value\_of(sigma\_vec[n]);

200

201  // Compute

202  const double Pn = 1.0 / ( 1.0 + exp( - (y\_dbl - mu\_dbl)

203  \* sigma\_inv\_vec ) );

204

205  P \*= Pn;

206

207  if (!is\_constant\_struct<T\_y>::value)

208  operands\_and\_partials.d\_x1[n]

209  += exp(logistic\_log(y\_dbl, mu\_dbl, sigma\_dbl)) / Pn;

210  if (!is\_constant\_struct<T\_loc>::value)

211  operands\_and\_partials.d\_x2[n]

212  += - exp(logistic\_log(y\_dbl, mu\_dbl, sigma\_dbl)) / Pn;

213  if (!is\_constant\_struct<T\_scale>::value)

214  operands\_and\_partials.d\_x3[n] += - (y\_dbl - mu\_dbl) \* sigma\_inv\_vec

215  \* exp(logistic\_log(y\_dbl, mu\_dbl, sigma\_dbl)) / Pn;

216  }

217

218  if (!is\_constant\_struct<T\_y>::value) {

219  for(size\_t n = 0; n < stan::length(y); ++n)

220  operands\_and\_partials.d\_x1[n] \*= P;

221  }

222  if (!is\_constant\_struct<T\_loc>::value) {

223  for(size\_t n = 0; n < stan::length(mu); ++n)

224  operands\_and\_partials.d\_x2[n] \*= P;

225  }

226  if (!is\_constant\_struct<T\_scale>::value) {

227  for(size\_t n = 0; n < stan::length(sigma); ++n)

228  operands\_and\_partials.d\_x3[n] \*= P;

229  }

230

231  return operands\_and\_partials.to\_var(P);

232

233  }

234

235  template <typename T\_y, typename T\_loc, typename T\_scale>

236  typename return\_type<T\_y, T\_loc, T\_scale>::type

237  logistic\_cdf\_log(const T\_y& y, const T\_loc& mu, const T\_scale& sigma) {

238

239  // Size checks

240  if ( !( stan::length(y) && stan::length(mu) && stan::length(sigma) ) )

241  return 0.0;

242

243  // Error checks

244  static const char\* function = "stan::prob::logistic\_cdf\_log(%1%)";

245

246  using stan::math::check\_not\_nan;

247  using stan::math::check\_positive\_finite;

248  using stan::math::check\_finite;

249  using stan::math::check\_consistent\_sizes;

250  using stan::math::value\_of;

251  using boost::math::tools::promote\_args;

252

253  double P(0.0);

254

255  check\_not\_nan(function, y, "Random variable", &P);

256  check\_finite(function, mu, "Location parameter", &P);

257  check\_positive\_finite(function, sigma, "Scale parameter", &P);

258  check\_consistent\_sizes(function, y, mu, sigma,

259  "Random variable", "Location parameter",

260  "Scale parameter", &P);

261

262  // Wrap arguments in vectors

263  VectorView<const T\_y> y\_vec(y);

264  VectorView<const T\_loc> mu\_vec(mu);

265  VectorView<const T\_scale> sigma\_vec(sigma);

266  size\_t N = max\_size(y, mu, sigma);

267

268  agrad::OperandsAndPartials<T\_y, T\_loc, T\_scale>

269  operands\_and\_partials(y, mu, sigma);

270

271  // Explicit return for extreme values

272  // The gradients are technically ill-defined, but treated as zero

273

274  for (size\_t i = 0; i < stan::length(y); i++) {

275  if (value\_of(y\_vec[i]) == -std::numeric\_limits<double>::infinity())

276  return operands\_and\_partials.to\_var(-std::numeric\_limits<double>::infinity());

277  }

278

279  // Compute vectorized cdf\_log and its gradients

280  for (size\_t n = 0; n < N; n++) {

281

282  // Explicit results for extreme values

283  // The gradients are technically ill-defined, but treated as zero

284  if (value\_of(y\_vec[n]) == std::numeric\_limits<double>::infinity()) {

285  continue;

286  }

287

288  // Pull out values

289  const double y\_dbl = value\_of(y\_vec[n]);

290  const double mu\_dbl = value\_of(mu\_vec[n]);

291  const double sigma\_dbl = value\_of(sigma\_vec[n]);

292  const double sigma\_inv\_vec = 1.0 / value\_of(sigma\_vec[n]);

293

294  // Compute

295  const double Pn = 1.0 / ( 1.0 + exp( - (y\_dbl - mu\_dbl)

296  \* sigma\_inv\_vec ) );

297  P += log(Pn);

298

299  if (!is\_constant\_struct<T\_y>::value)

300  operands\_and\_partials.d\_x1[n]

301  += exp(logistic\_log(y\_dbl, mu\_dbl, sigma\_dbl)) / Pn;

302  if (!is\_constant\_struct<T\_loc>::value)

303  operands\_and\_partials.d\_x2[n]

304  += - exp(logistic\_log(y\_dbl, mu\_dbl, sigma\_dbl)) / Pn;

305  if (!is\_constant\_struct<T\_scale>::value)

306  operands\_and\_partials.d\_x3[n] += - (y\_dbl - mu\_dbl) \* sigma\_inv\_vec

307  \* exp(logistic\_log(y\_dbl, mu\_dbl, sigma\_dbl)) / Pn;

308  }

309

310  return operands\_and\_partials.to\_var(P);

311  }

312

313  template <typename T\_y, typename T\_loc, typename T\_scale>

314  typename return\_type<T\_y, T\_loc, T\_scale>::type

315  logistic\_ccdf\_log(const T\_y& y, const T\_loc& mu, const T\_scale& sigma) {

316

317  // Size checks

318  if ( !( stan::length(y) && stan::length(mu) && stan::length(sigma) ) )

319  return 0.0;

320

321  // Error checks

322  static const char\* function = "stan::prob::logistic\_cdf\_log(%1%)";

323

324  using stan::math::check\_not\_nan;

325  using stan::math::check\_positive\_finite;

326  using stan::math::check\_finite;

327  using stan::math::check\_consistent\_sizes;

328  using stan::math::value\_of;

329  using boost::math::tools::promote\_args;

330

331  double P(0.0);

332

333  check\_not\_nan(function, y, "Random variable", &P);

334  check\_finite(function, mu, "Location parameter", &P);

335  check\_positive\_finite(function, sigma, "Scale parameter", &P);

336  check\_consistent\_sizes(function, y, mu, sigma,

337  "Random variable", "Location parameter",

338  "Scale parameter", &P);

339

340  // Wrap arguments in vectors

341  VectorView<const T\_y> y\_vec(y);

342  VectorView<const T\_loc> mu\_vec(mu);

343  VectorView<const T\_scale> sigma\_vec(sigma);

344  size\_t N = max\_size(y, mu, sigma);

345

346  agrad::OperandsAndPartials<T\_y, T\_loc, T\_scale>

347  operands\_and\_partials(y, mu, sigma);

348

349  // Explicit return for extreme values

350  // The gradients are technically ill-defined, but treated as zero

351

352  for (size\_t i = 0; i < stan::length(y); i++) {

353  if (value\_of(y\_vec[i]) == -std::numeric\_limits<double>::infinity())

354  return operands\_and\_partials.to\_var(0.0);

355  }

356

357  // Compute vectorized cdf\_log and its gradients

358  for (size\_t n = 0; n < N; n++) {

359

360  // Explicit results for extreme values

361  // The gradients are technically ill-defined, but treated as zero

362  if (value\_of(y\_vec[n]) == std::numeric\_limits<double>::infinity()) {

363  return operands\_and\_partials.to\_var(stan::math::negative\_infinity());

364  }

365

366  // Pull out values

367  const double y\_dbl = value\_of(y\_vec[n]);

368  const double mu\_dbl = value\_of(mu\_vec[n]);

369  const double sigma\_dbl = value\_of(sigma\_vec[n]);

370  const double sigma\_inv\_vec = 1.0 / value\_of(sigma\_vec[n]);

371

372  // Compute

373  const double Pn = 1.0 - 1.0 / ( 1.0 + exp( - (y\_dbl - mu\_dbl)

374  \* sigma\_inv\_vec ) );

375  P += log(Pn);

376

377  if (!is\_constant\_struct<T\_y>::value)

378  operands\_and\_partials.d\_x1[n]

379  -= exp(logistic\_log(y\_dbl, mu\_dbl, sigma\_dbl)) / Pn;

380  if (!is\_constant\_struct<T\_loc>::value)

381  operands\_and\_partials.d\_x2[n]

382  -= - exp(logistic\_log(y\_dbl, mu\_dbl, sigma\_dbl)) / Pn;

383  if (!is\_constant\_struct<T\_scale>::value)

384  operands\_and\_partials.d\_x3[n] -= - (y\_dbl - mu\_dbl) \* sigma\_inv\_vec

385  \* exp(logistic\_log(y\_dbl, mu\_dbl, sigma\_dbl)) / Pn;

386  }

387

388  return operands\_and\_partials.to\_var(P);

389  }

390

391  template <class RNG>

392  inline double

393  logistic\_rng(const double mu,

394  const double sigma,

395  RNG& rng) {

396  using boost::variate\_generator;

397  using boost::random::exponential\_distribution;

398

399  static const char\* function = "stan::prob::logistic\_rng(%1%)";

400

401  using stan::math::check\_positive\_finite;

402  using stan::math::check\_finite;

403

404  check\_finite(function, mu, "Location parameter", (double\*)0);

405  check\_positive\_finite(function, sigma, "Scale parameter", (double\*)0);

406

407  variate\_generator<RNG&, exponential\_distribution<> >

408  exp\_rng(rng, exponential\_distribution<>(1));

409  return mu - sigma \* std::log(exp\_rng() / exp\_rng());

410  }

411  }

412 }

413 #endif

check\_not\_nan.hpp

stan::prob::logistic\_cdf\_log

return\_type< T\_y, T\_loc, T\_scale >::type logistic\_cdf\_log(const T\_y &y, const T\_loc &mu, const T\_scale &sigma)

**Definition:** logistic.hpp:237

traits.hpp

stan::math::log1p

boost::math::tools::promote\_args< T >::type log1p(const T x)

Return the natural logarithm of one plus the specified value.

**Definition:** log1p.hpp:39

check\_positive\_finite.hpp

stan::agrad::OperandsAndPartials::to\_var

T\_return\_type to\_var(double logp)

**Definition:** partials\_vari.hpp:138

stan::math::check\_positive\_finite

bool check\_positive\_finite(const char \*function, const T\_y &y, const char \*name, T\_result \*result)

**Definition:** check\_positive\_finite.hpp:12

stan::length

size\_t length(const T &)

**Definition:** traits.hpp:159

stan::DoubleVectorView

DoubleVectorView allocates double values to be used as intermediate values.

**Definition:** traits.hpp:358

stan::math::check\_finite

bool check\_finite(const char \*function, const T\_y &y, const char \*name, T\_result \*result)

Checks if the variable y is finite.

**Definition:** check\_finite.hpp:52

stan::prob::logistic\_log

return\_type< T\_y, T\_loc, T\_scale >::type logistic\_log(const T\_y &y, const T\_loc &mu, const T\_scale &sigma)

**Definition:** logistic.hpp:27

partials\_vari.hpp

stan::agrad::value\_of

T value\_of(const fvar< T > &v)

Return the value of the specified variable.

**Definition:** value\_of.hpp:16

constants.hpp

stan::is\_vector

**Definition:** traits.hpp:83

stan::prob::logistic\_ccdf\_log

return\_type< T\_y, T\_loc, T\_scale >::type logistic\_ccdf\_log(const T\_y &y, const T\_loc &mu, const T\_scale &sigma)

**Definition:** logistic.hpp:315

stan::agrad::OperandsAndPartials

A variable implementation that stores operands and derivatives with respect to the variable...

**Definition:** partials\_vari.hpp:76

traits.hpp

stan::return\_type::type

boost::math::tools::promote\_args< typename scalar\_type< T1 >::type, typename scalar\_type< T2 >::type, typename scalar\_type< T3 >::type, typename scalar\_type< T4 >::type, typename scalar\_type< T5 >::type, typename scalar\_type< T6 >::type >::type type

**Definition:** traits.hpp:406

stan::is\_constant\_struct

Metaprogram to determine if a type has a base scalar type that can be assigned to type double...

**Definition:** traits.hpp:57

stan::math::value\_of

double value\_of(const T x)

Return the value of the specified scalar argument converted to a double value.

**Definition:** value\_of.hpp:24

stan::prob::include\_summand

Template metaprogram to calculate whether a summand needs to be included in a proportional (log) prob...

**Definition:** traits.hpp:35

stan::agrad::OperandsAndPartials::d\_x2

VectorView< double \*, is\_vector< T2 >::value, is\_constant\_struct< T2 >::value > d\_x2

**Definition:** partials\_vari.hpp:83

stan::math::check\_consistent\_sizes

bool check\_consistent\_sizes(const char \*function, const T1 &x1, const T2 &x2, const char \*name1, const char \*name2, T\_result \*result)

**Definition:** check\_consistent\_sizes.hpp:13

stan::max\_size

size\_t max\_size(const T1 &x1, const T2 &x2)

**Definition:** traits.hpp:191

log1p.hpp

stan::prob::logistic\_rng

double logistic\_rng(const double mu, const double sigma, RNG &rng)

**Definition:** logistic.hpp:393

stan::math::check\_not\_nan

bool check\_not\_nan(const char \*function, const T\_y &y, const char \*name, T\_result \*result)

Checks if the variable y is nan.

**Definition:** check\_not\_nan.hpp:56

check\_finite.hpp

value\_of.hpp

stan::agrad::OperandsAndPartials::d\_x1

VectorView< double \*, is\_vector< T1 >::value, is\_constant\_struct< T1 >::value > d\_x1

**Definition:** partials\_vari.hpp:82

stan::agrad::OperandsAndPartials::d\_x3

VectorView< double \*, is\_vector< T3 >::value, is\_constant\_struct< T3 >::value > d\_x3

**Definition:** partials\_vari.hpp:84

constants.hpp

stan::agrad::log

fvar< T > log(const fvar< T > &x)

**Definition:** log.hpp:15

stan::VectorView

VectorView is a template metaprogram that takes its argument and allows it to be used like a vector...

**Definition:** traits.hpp:275

stan::agrad::exp

fvar< T > exp(const fvar< T > &x)

**Definition:** exp.hpp:16

check\_consistent\_sizes.hpp

stan::agrad::log1p

fvar< T > log1p(const fvar< T > &x)

**Definition:** log1p.hpp:16

stan::math::negative\_infinity

double negative\_infinity()

Return negative infinity.

**Definition:** constants.hpp:123

stan::prob::logistic\_cdf

return\_type< T\_y, T\_loc, T\_scale >::type logistic\_cdf(const T\_y &y, const T\_loc &mu, const T\_scale &sigma)

**Definition:** logistic.hpp:143


---

[ Stan Home Page ]

*© 2011–2014,
Stan Development Team.*
